# Fault Recognition Method Based on Attention Mechanism and the 3D-UNet

**DOI:** 10.1155/2022/9856669

**Published:** 2022-04-21

**Authors:** Ting Yu, Xin Wang, Tong Jun Chen, Chang Wei Ding

**Affiliations:** ^1^School of Computer Science and Technology, China University of Mining and Technology, Xu'zhou 221000, China; ^2^School of Earth and Geoscience, China University of Mining and Technology, Xu'zhou 221000, China

## Abstract

Oil and gas reservoirs are of great significance for economic benefits. Faults act as important conduits for transporting hydrocarbons and as essential sealing conditions. The location and morphology of faults reflect changes in the shape of the strata, so fault interpretation of seismic data has been an essential task in oil and gas exploration and development. The traditional fault identification method is time-consuming and inaccurate with large uncertainties. This paper proposed a fault recognition method based on 3D-UNet that added attention mechanisms to a convolutional neural network (CNN). This approach takes advantage of the UNet end-to-end architecture and the attention mechanism to focus on essential areas and suppress irrelevant information, allowing the model to focus on more valuable features. A fault identification network for seismic data was proposed by combining the 3D-UNet architecture with the Squeeze-and-Excitation (SE) attention mechanism. The 3D-UNet architecture comprises two parts: encoding and decoding, and the network architecture realizes end-to-end training. At the same time, SE was used to focus on the advantages of feature channels, further improving the accuracy of the network. The model performance was evaluated using the synthetic dataset provided by Wu. Experimental results show that the proposed model has better prediction performance in terms of accuracy, better recognition continuity, and richer fault detail.

## 1. Introduction

Faults are one of the fundamental geological phenomena. With the development of oil and gas from the shallow formation to the deep formation, faults are the main path of oil and gas migration or underground sealing structures for oil, gas, and water. To speed up the exploration and development, people hope the interpretation speed and precision are as high as possible. Therefore, promoting efficiency and improving productivity become the most critical issues in modern seismic exploration. As a result, fault identification methods have evolved from conventional manual fault interpretation methods to artificial intelligence methods.

After seismic data processing, the conventional artificial fault interpretation method distinguishes the discontinuities of a seismic event in the profile. Although this method can accurately identify faults, it relies too much on the subjective awareness of fault interpreters, which is a heavy and time-consuming workload. In order to overcome the shortcomings in manual fault identification methods, many scholars have conducted extensive research to speed up fault identification, which is mainly carried out by calculating seismic data attributes. Some scholars calculated the semblance [1], coherency [2, 3], variance [4], and gradient magnitude [5] from seismic data to assist fault interpretation. Although the interpretation efficiency was greatly improved, there may be some noise in the extracted seismic attributes, so the identification accuracy of faults is relatively poor. Subsequent related work still requires manual intervention.

Researchers have proposed automatic or semiautomatic techniques to reduce the identification time further for faults. For example, the proposed ant tracking algorithm [6, 7] and optimal surface voting technology [8] have further enhanced seismic attributes while suppressing noise, increasing fault identification accuracy and speed. Jacquemin and Mallet [9] proposed a double hough transform to detect faults automatically. Yan et al. [10] proposed combining principal component analysis (PCA) to realize forward diffusion and backward diffusion to enhance fault features. These methods can extract fault features well, but the fault interpretation speed is slow and not all faults can be detected.

With the development of machine learning [11–14], researchers have combined seismic attributes and machine learning algorithms to identify faults automatically. Di et al. [15] used seismic attributes to highlight faults, create a dataset, and conduct a support vector machine (SVM) analysis on the data set to detect faults. Tingdahl and de Rooij [16] used various seismic attributes as the input of artificial neural networks to identify and predict faults. These methods are computationally intensive and noise sensitive and do not consider the spatial characteristics of the fault distribution. Recently, deep learning has made remarkable achievements, and many scholars further use deep learning to solve the problem of fault identification. Wu et al. [17] proposed a faulted network model based on UNet end-to-end model. Seismic data samples were taken as the input of the neural network model to predict faults by learning fault characteristics. Xiong et al. [18] used slices in three directions as three-channel input of the neural network to predict the intersection of pieces in three principles to judge whether it was a fault. Chang et al. [19] used a residual neural network to establish the UNet model for fault detection and carried out multiscale and multilevel feature extraction of seismic data and deep learning method for fault recognition with high accuracy and low loss rate, ensuring the accuracy of fault recognition.

Because many of the above deep learning uses the UNet structure of encoding and decoding, it shows that UNet has a good performance in seismic fault identification and provides a new solution for seismic fault identification. In this paper, we transformed the fault identification problem into a binary image segmentation problem; that is, 1 represents faults, and 0 represents nonfault. The 3D-UNet architecture is used as the base model, and an attention module is added (see Section 3). The main contribution of this paper is to design a network model that adds an attention mechanism. The SE module can be easily implemented and embedded into the 3D-UNet model, and it can be trained end-to-end to obtain the fault prediction model and realize automatic fault identification. In the absence of dimensionality reduction, the SE module captures channel attention through feature channels to assign high importance scores to fault-related features, which are used to increase attention to fault areas. Our proposed method performs well on the synthetic seismic dataset provided by Wu.

This paper is organized as follows. A brief overview of related research on attention mechanisms is presented in Section 2. Subsequently, we describe the proposed approach and other models for prediction in Section 3.  Section 4 presents the experimental results and analysis. Lastly, we end with some conclusive remarks in Section 5.

## 2. Related Work

Since the AlexNet [20] network achieved good results in the image classification challenge in 2012, many methods have been proposed to deepen the performance of the convolutional network. Some work adapts structure from the connection mode of the convolution layer, pooling layer, and complete connection layer [21, 22], and some work focuses on the deeper network [23, 24]. These models further improve the performance of convolutional networks in terms of width and depth. In 2015, Ronneberger et al. [25] proposed UNet to solve the image segmentation problem in the medical field. UNet is mainly divided into encoding and decoding, an asymmetric structure. It uses skip connections to concatenate the outputs of each layer of the decoder with the inputs of each layer of the upsampling so that the low-level features can be better expressed. As inspired by UNet, Zhou et al. [26] proposed UNet++. This model can be regarded as a combination of many small UNet stacked by many units. In the final test, the pruning algorithm removes redundant connections. The difference between 3D-UNet [27] and 2D UNet lies in that 2D operation is transformed into 3D operation, but the basic structure remains unchanged. 3D images are not converted into slices and then input into a 2D network, but the whole picture can be input into the network model as data. Compared with UNet, Vnet is a variant of UNet. Vnet [28] is proposed for 3D data, so the final output is 3D data of single channel and a short connection mode is added in each stage, which also realizes end-to-end training. Since seismic fault data have a *Z*-axis, which is equivalent to a cube, 3D convolution is used in the cube to obtain the feature map. The disadvantage is that the number of three-dimensional convolution parameters is large, it is difficult to train, and it cannot transfer learning with the pretrained model.

The attention mechanism is derived from the study of human vision. Humans will selectively focus on the area of interest in an image and then devote much attention to this area while ignoring other places to obtain more details of the target to be paid attention to and suppress irrelevant information [29–31]. Recently, the combination of CNNs and attention mechanisms has been effectively applied to many fields. For example, Trebing et al. [32] designed SmaAt-UNet which combines the attention mechanism of UNet and convolution block attention module (CBAM) into the prediction of the weather forecast, Li et al. [33] proposed to use the attention mechanism to establish the connection between feature images and combine lightweight attention and UNet for segmentation of retinal vessel images, and both of them achieved good results. Attention value calculation identifies the critical areas in the image through a new layer of weight parameters so that the neural network can learn the crucial information of each picture. Then, the network can automatically adjust the weight to adapt to different recognition tasks. Its essence is to locate exciting information and suppress useless information to quickly screen out more valuable information for the current study from a large amount of information and improve the feature extraction ability. Therefore, the core point of the attention mechanism lies in how to calculate attention. Scholars have performed many kinds of research on calculating attention value, mainly from the three directions of channel attention, spatial attention, and channel and space mixed engagement. Hu et al. [34] proposed that the innovation of the SE module lies in its focus on the relationship between media because the convolution operation of the original CNN model is to multiply the feature information in the convolution region and the convolution kernel and then add them to obtain the new channel information. The squeeze-excitation operation scores each channel feature, making the network pay more attention to the most informative channel features while suppressing the less important ones. The FC layer used in SE for dimensionality reduction has a side effect on the attention mechanism, and the dependencies between channels are not high. Therefore, ECA [35] module gives up dimensionality reduction and considers each channel and *k* neighbors to achieve appropriate channel interaction. ECA uses Conv1D after global average pooling, and the local cross-channel interaction rate, namely, *K*, is realized by the convolution kernel size of one-dimensional convolution.

In contrast, the interaction rate adopts a self-convolution adaptation algorithm. That is, the convolution kernel size varies with the number of channels. The ECA module is further lighter and has fewer parameters than SE. Since SE and ECA modules only calculate the attention of the features between tracks, they still consider the spatial features equal weight, so they do not calculate the importance of the spatial features. The spatial feature attention mechanism is just the opposite of the SE module. It calculates the weight of spatial features and considers the weight of channel features to be the same. Therefore, Woo et al. [36] proposed the CBAM, which combines spatial and channel elements to calculate attention. CBAM comprises two modules, Channel Attention Module (CAM) and Spatial Attention Module (SAM). CAM first adopts max-pooling and average pooling operations and then obtains feature maps through shared multilayer perceptron (MLP). We carried out the max-pooling and average pooling operations in parallel; we got the feature maps of channel attention by adding the two feature maps and multiplying the original feature maps after the sigmoid function. The input of spatial attention is the feature map of channel attention and carries out max-pooling and average pooling. Then, the two feature maps are spliced together for convolution operation. After the same sigmoid, we got the new feature map by multiplying the original feature map. CAM is equivalent to adding max-pooling in the SE module. CBAM proves that global max-pooling and average pooling can improve efficiency.

## 3. Method

### 3.1. Proposed Network

**Figure 1:**
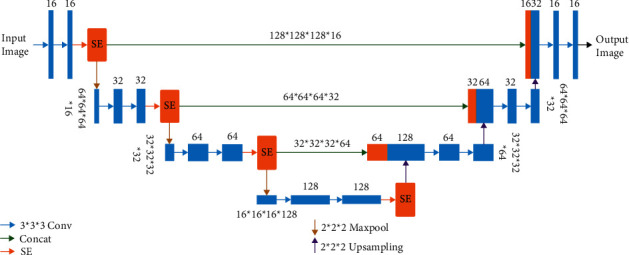
An example of an input fed through our proposed model.

We propose a network architecture combining 3D-UNet and SE modules. The model we proposed is a 3D-UNet network architecture established and extended, as shown in Figure 1. The original UNet comprises four downsampling layers and upsampling layers. This design has reduced the layers of downsampling layer and upsampling layer into three due to GPU limitations. The core function of the downsampling layer is to extract context information to facilitate better the classification of the classifier. Each downsampling layer will further extract features for advanced feature services. The function of the upsampling layer is to double the original size. The 3D-UNet network presents a U-shaped structure composed of encoding and decoding parts. In the coding part on the left, each operation contains two convolution blocks, the size of the convolution kernel is 3 ∗ 3 ∗ 3, and then our SE module is next, and the SE receives the feature map on the left. The pooling operation is connected to the attention module output, and max-pooling is used to compare parameters further and extract compelling features. The pooling step size is 2 ∗ 2 ∗ 2. After the encoding part is over, the corresponding decoding part follows. The decoding amount is the same as the encoding amount having three upsampling layers. The skip connection operation splices the low-level semantic information with the high-level semantic information to capture more helpful information (the innovation of the UNet network). The attention output of each layer of the coding part is concatenated with the corresponding upsampling output and then uses two convolution operations to halve the feature channel. The size of the convolution kernel is also 3 ∗ 3 ∗ 3. The output of the last layer is a 1 ∗ 1 ∗ 1 convolution operation and sigmoid function so that the value of each pixel in the final output image corresponds to within 0-1. Finally, the output size of the network model is consistent with the input size.

The principle of the SENet module is to distinguish according to the importance of each channel. The global average pooling first compresses the spatial dimension for each output channel, and each channel obtains one scalar. We squeezed the global feature into a number with the international receptive field. We got the number of *C* channels, and the size is changed from *H* ∗ *W* ∗ *C* to 1 ∗ 1 ∗ *C* so that the output dimension and the number of input feature channels can be kept consistent. The second is to pass the global features through a structure such as FC-ReLU-FC-Sigmoid to obtain *C* scalars between 0 and 1. The first FC layer reduces the dimensionality, reducing the feature dimension to 1/*r* of the input. The other FC layer is used to return to the original feature dimension. The FC layer and Relu activation function can reduce parameters and improve nonlinearity. Finally, we use the sigmoid function to get *C* values between 0 and 1, which is the weight of each channel. Then, we multiply the original feature map to get a new feature map. The first step is global average pooling, called squeeze. Then, the two FC layers and Relu activation functions are used to obtain a weight value between 0 and 1. Then, we use a fully connected neural network to perform a nonlinear transformation on the result after the squeeze operation, called excitation. Therefore, the squeeze-excitation operation is equivalent to scoring each channel feature. The network pays great attention to those channel features with a large amount of information while suppressing relatively unimportant parts.

**Figure 2:**
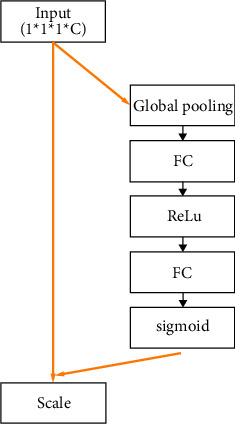
SE Module flow chart.

Since the SE module is proposed for two-dimensional data, the original module needs to be modified while the seismic data is three-dimensional. The frame diagram of the SE module is shown in Figure 2. The first is the squeeze operation. That is, through a global average pooling operation, *H* ∗ *W* ∗ *Z* ∗ *C* becomes a scalar of 1 ∗ 1 ∗ 1 ∗ *C*, and the formula is as follows:

(1) $$\begin{array}{c} z_{c}=F_{sq}\left(u_{c}\right)=\frac{1}{X*Y*Z}\sum_{i=1}^{X}\sum_{j=1}^{Y}\sum_{t=1}^{Z}u_{c}\left(i,j,t\right),\;z{ɛ\text{R}}^{c}, \end{array}$$

where *u*_*c*_  represents the original feature, and *X*, *Y*, and *Z* represent the seismic input size.

Next is the excitation operation, as shown in formula (2), multiplying W1 by *z* is equivalent to the first FC layer. The dimension of *W*_1_ is C/*r* ∗C, and *r* is the scaling parameter. We set it to 8 in this article and reduced the parameters through this parameter *r*. Therefore, the result of *W*_1_ multiplied by *z* is 1∗1∗1∗C/*r*, and the output dimension remains unchanged. Then, multiplying *W*_2_ by the output result of the activation function is equivalent to the second layer of the FC layer, and the dimension of *W*_2_ is C∗C/*r*. So the output dimension is 1∗1∗1∗C, and through the sigmoid function, C scalars between 0 and 1 are obtained finally, and the dimension of *s* is 1∗1∗1∗C.

(2) $$\begin{array}{c} s=F_{ex}\left(z,W\right)=\sigma \left(g\left(z,W\right)\right)=\sigma \left(W_{2}\delta \left(W_{1}z\right)\right), \end{array}$$

where *z* is the result obtained by squeezing, *δ* is the Relu activation function, *σ* is the sigmoid function, and *s* is the weight of each channel.

Finally, the weight is weighted to the original feature by multiplication, and the formula is as follows:

(3) $$\begin{array}{c} \tilde{X_{c}}=F_{\text{scale}}\left(u_{c},s_{c}\right)=u_{c}*\;s_{c}. \end{array}$$

The numbers above each bar display the number of channels; the sheer numbers on the left and right sides correspond to the input size.

### 3.2. Other Networks

For comparison, we also trained other network structures based on 3D-UNet with two different attention mechanisms, CBAM and ECA. They are the proposed models and 3D-UNet, 3D-UNet with CBAM, and 3D-UNet and ECA models.  Table 1 shows the training parameters of each attention mechanism. Since the attention mechanism is only added to UNet, the complexity of all models is the same. These models are implemented in the platform of Keras, where we used a 3 ∗ 3 ∗ 3 convolution kernel for the convolution layer and a 2 ∗ 2 ∗ 2 max-pooling step for the pooling layer. Comparing the classical image segmentation model of a fully convolution network (FCN) and attention gate added AttUNet with our proposed model, the FCN model stride is 4 and 8.  Table 2 shows the number of parameters and model complexity of the image segmentation model. FLOPs are calculated with the input resolution of a 128 ∗ 128 ∗ 128 size.

## 4. Experiment

### 4.1. Data

**Figure 3:**
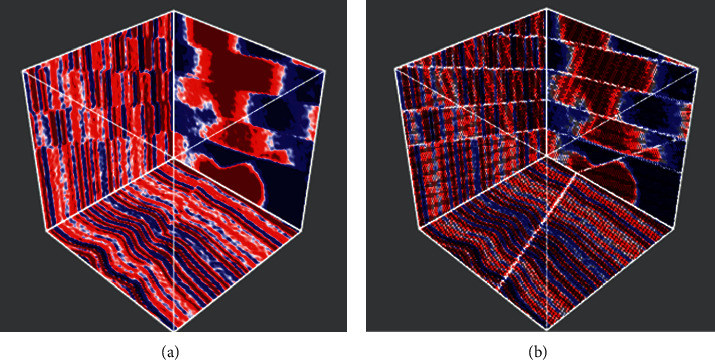
A seismic data set (a) and its corresponding binary fault labeling data set (b) with the size of 128 ∗ 128 ∗ 128.

**Figure 4:**
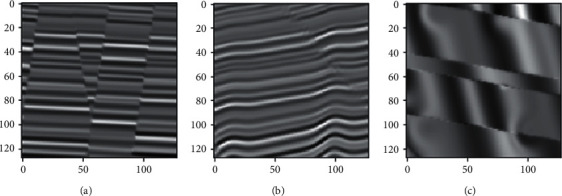
Seismic slices along with Inline direction (a), Xline direction (b), and Time direction (c).

This paper uses the public dataset provided by Wu to test, which was simulated based on the classical convolution model theory. Firstly, an initial horizontal reflectance model with transverse convolution variation is generated by a modified two-dimensional Gaussian formulation. Secondly, to increase the complexity of the model, a new flat reflectance model is obtained by adding plane shear stress to the initial horizontal reflectance model. In addition, faults with different strikes, dips, throws, and displacements are added to the geophysical model, and a Ricker wavelet is convoluted with the final reflectivity model to obtain the synthetic seismic data. Finally, a certain amount of random noise is added to the simulated seismic data to improve the realism of the synthetic seismic records [39]. The dataset contains 200 training samples and 20 verification samples. Each collection of samples contains seismic data and their corresponding fault labels, with a size of 128 ∗ 128 ∗ 128, as shown in Figure 3.  Figure 4 depicts slices of selected planes in the Inline, Xline, and Time directions. The Inline direction is generally the main survey line, perpendicular to the geological structures and evenly arranged, so the fault is more visible in the Inline slice.

### 4.2. Train

#### 4.2.1. Hyperparameter Settings

The models mentioned above are trained for 25 epochs, where the Adam optimizer was used [40], and the initial learning rate was set to l*e* − 4. The hardware used for the training process is four Tesla V100-SXM2-32GB graphics cards. We input 3D seismic images into the network in a batch each time. Each batch includes the original image and its rotated images after 90°, 180°, and 270° rotations. The image size of seismic data is 128 ∗ 128 ∗ 128.

#### 4.2.2. Model Evaluation

Due to the imbalanced distribution of fault and nonfault samples in the dataset, we use the Balanced Cross-Entropy Loss Function proposed by Xie and Tu [41]:

(4) $$\begin{array}{c} L.=.e\beta \sum_{i=0}^{i=N}y_{i}\log\left(p_{i}\right)-\;\left(1+\beta \right)\cdot \sum_{i=0}^{i=N}\left(1-y_{i}\right)\log\left(1-p_{i}\right). \end{array}$$

The loss function uses *β* =  ∑_*i*=0_^*i*=*N*^(1 − *y*_*i*_)/*N* to represent the proportion of pixels occupied by the nonfault in the entire picture, and 1 − *β* represents the proportion of pixels occupied by the fault in the whole 3D seismic image. To distinguish whether it is a fault or a nonfault in the pixel-level 3D image, we use precision-recall (PRC) [42] and receiver-operating-characteristic (ROC) [43] curves to evaluate the pixel-level classification result and the performance of the classifier. As the horizontal and vertical coordinates show that the distributions of true-positive rate (TPR) and false-positive rate (FPR) of samples are not balanced, the ROC curve will be a more stable indicator that can reflect the quality of the model. If the number of negative samples increases, false positives (FP) and true negatives (TN) will increase. The PRC's horizontal and vertical coordinates will be affected so that the PRC curve will change accordingly. However, comparing the ROC curve calculation formula, when the number of negative samples increases, FP and TN will increase proportionally, so their values will not be affected and will not be affected. The PRC curve is convex on the right, the better. The ROC curve is convex on the left, the better. Because we wanted to analyze the model quantitatively, we calculated the AUC area (the area under the ROC curve). The larger the AUC area, the better the model performance. The F-1 score is the harmonic mean of precision and recall. Only when precision and recall are both good, the F-1 score can be large enough. The definitions are as follows:

(5) $$\begin{array}{c} \text{FPR}=\frac{\text{FP}}{\text{FP}+\text{TN}},\\ \text{TPR}=\frac{\text{TP}}{\text{TP}+\text{FN}},\\ \text{Precision}=\frac{\text{TP}}{\text{FP}+\text{TP}},\\ \text{Recall}=\frac{\text{TP}}{\text{FN}+\text{TP}},\\ \text{F}-1=\frac{2\mathrm{*Precision*Recall}}{\text{Precision}+\text{Recall}}. \end{array}$$

## 5. Results and Discussion

**Figure 5:**
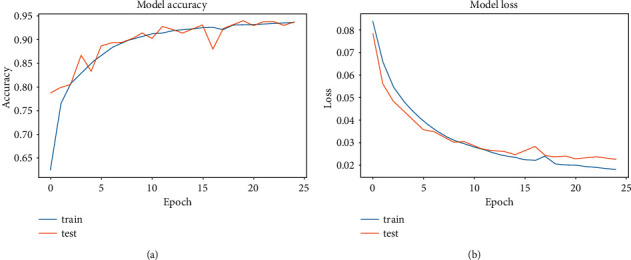
Model accuracies (a) and losses (b) of training and validation sets for the proposed model.

After training the four models discussed in Section 3, we select the model with the minor verification loss for each model from the training process and then test and evaluate it on the verification set.  Figure 5(a) is the model accuracy curve of the training and test sets, and it shows that the accuracy of our proposed model is stable at about 94% after 25 epochs.  Figure 5(b) shows the loss function curves of the training and test sets, and the value gradually converges to 0.01 at about 25 epochs.

**Figure 6:**
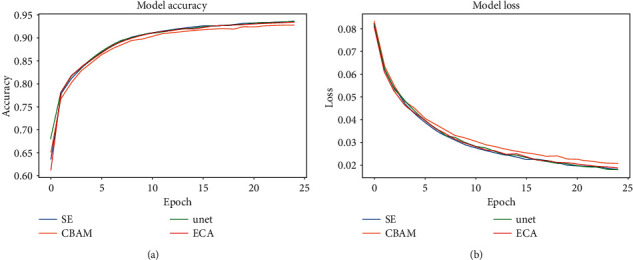
Accuracies (a) and losses (b) of the four models.

Comparing the proposed model results with the other models, we find that all models gradually reached about 92% accuracy and 0.02 loss after 25 epochs, and the proposed model (blue line) has the highest accuracy and fast convergence rate among all models, as shown in Figure 6. The accuracy rate of the 3D-UNet with the ECA module is slightly lower than that of the SE module but higher than that of the other two attention modules. Since the CBAM attention mechanism combines the attention of both space and channel, our proposed model is more accurate than the 3D-UNet with the CBAM module. On the one hand, the performance of the 3D-UNet with the CBAM module is worse than that of the original 3D-UNet model (correct rates are 0.92 and 0.93, respectively). Although the proposed module has increased the number of parameters slightly, its accuracy is greatly improved compared with the CBAM module. The 3D-UNet and CBAM modules are slightly less accurate than other models. The proposed model has a faster convergence rate in all models, and the loss value drops faster. The 3D-UNet with the ECA module has a large loss value after the loss curve becomes stable, but the convergence speed is fast. Although the 3D-UNet model has a slower convergence rate than our proposed model, its loss continues to decrease after a certain learning period, the loss value is also small, and it is better than the 3D-UNet model with the CBAM module. The 3D-UNet model with the CBAM module converges slightly slower than other models.

**Figure 7:**
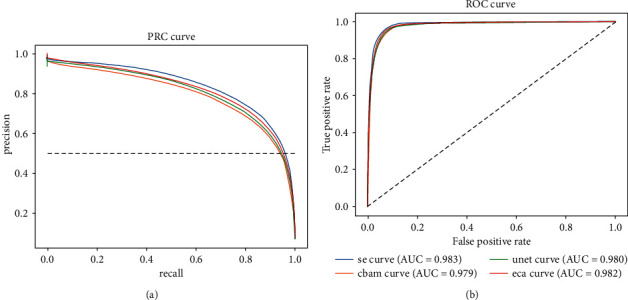
Precision-Recall (a) and ROC (b) curves of the four models.

In addition, we calculated the precision-recall and operating-characteristic curves of the model on the validation set, as shown in Figure 7. The model of UNet with SE module is at the top of the diagonal, and the model of 3D-UNet with CBAM module is at the bottom of the diagonal, so the classification effect of 3D-UNet with SE module is better. However, 3D-UNet with ECA module is the same as our proposed model. Since the gap between the ROC curves is small, we also compared the PRC curves, as shown in Figure 7(a). According to the PRC curve, 3D-UNet with SE module is at the top right, 3D-UNet with CBAM module is at the bottom right, and 3D-UNet with ECA module is at the top right of the other two models. Therefore, the classification effect of our proposed model is better.

We calculated the four evaluation indicators of the four models on the validation set and listed the scores in Table 3. This table shows that our proposed model performs best in most indicators. The lowest precision score belongs to 3D-UNet. The difference between the highest and lowest precision obtained by all four models on the data set is 0.116, while the lowest recall score belongs to the 3D-UNet with the ECA module. Since the precision and recall are adversarial, one of the two is high, and the other is low. Because the F-1 score is the average value of precision and recall, it can better reflect the quality of the model. The highest F-1 score belongs to the proposed model, and the lowest F-1 score belongs to 3D-UNet. The difference between the highest and the lowest is 0.096, which also shows that the SE attention we have added has played a significant role. As a larger AUC represents a better performance than the others, our proposed model has the highest score indicating the best performance.

When ranking the model, we can see that implementing 3D-UNet with SE performs best in almost all indicators. Although the model has an imbalance between the positive and negative samples of faults and nonfaults, the added SE attention helps fault recognition. The comprehensive comparison shows that the proposed model is better than other models regarding the accuracy, loss, and evaluation indicators. It also indicates that the proposed model has good performance in fault identification of seismic data.

The experiments compare the classical semantic segmentation model FCN and AttUNet with the addition of the attention gate with our proposed model. We calculated the evaluation metrics of the different segmentation models on the validation set and showed the results in Table 4.

As shown in Table 4, our proposed UNet-SE model has the best performance improvement among the models in terms of evaluation metrics. The FCN model has lower accuracy and F-1 score than the UNet model due to its disadvantage of combining context information. The difference between AttUNet and our proposed model is that it adds a gate, and although it has a certain degree of improvement in accuracy over the FCN model, the accuracy, AUC, and F1-score are all lower than the UNet-SE model.

**Figure 8:**
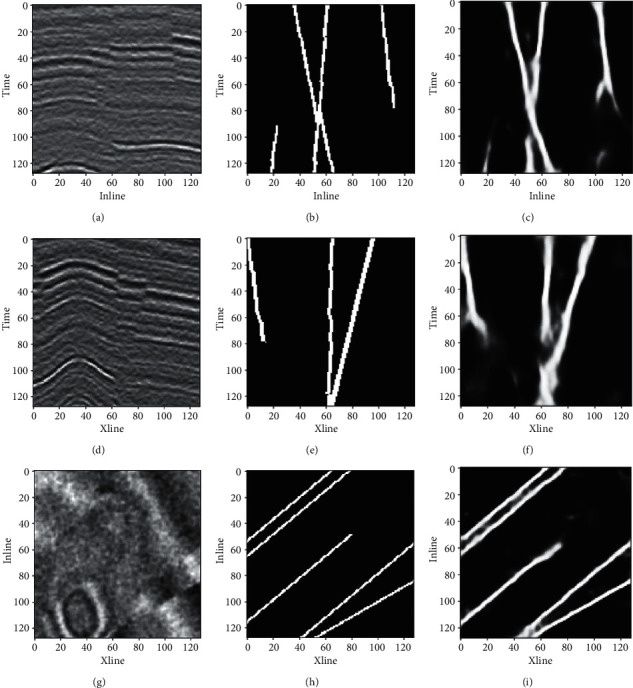
Fault recognition result for theoretical seismic data. (a) Xline slice of seismic data, (b) Xline slice with labeled faults, (c) Xline slice with predicted faults, (d) Inline slice of seismic data, (e) Inline slice with labeled faults, (f) Inline slice with predicted faults, (g) Time slice of seismic data, (h) Time slice with labeled faults, and (i) Time slice with predicted faults.

Theoretical data D013 (the 13th test sample) was used as an example to show the identification results of faults in slices along Xline, Inline, and Time directions, as shown in Figure 8. The shallow seismic events in the Xline slice show strong amplitude, large fault throw, and apparent discontinuity, and these features make fault identification easy. In contrast, the middle and deep seismic events in the Xline slice are weak in amplitude, blurring the faults. Therefore, it is challenging to identify the faults using manual interpretation or other identification methods through tracking seismic events and comparing their misalignment features. This paper uses 3D seismic data and fault label samples to establish the neural network that has learned the 3D characteristics of fault distribution and can identify faults with different occurrences and degrees of concealment. According to the figure, both the location and shape of the predicted faults are in line with the labeled real faults, which further verifies the excellent performance of the neural network model proposed in this paper regarding the accuracy and reliability of fault recognition.

**Figure 9:**
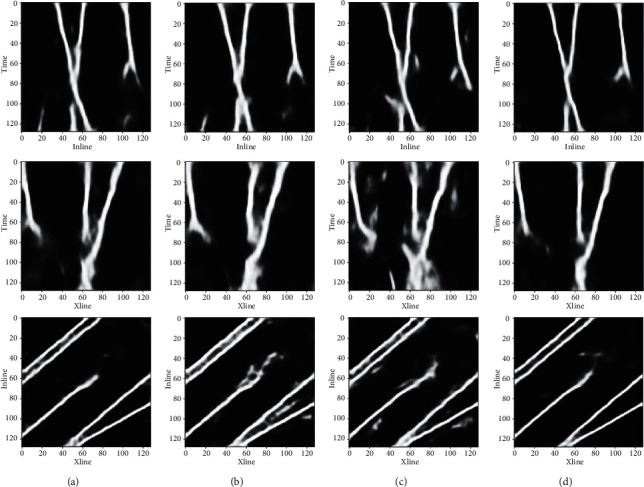
The recognition effect of different attention mechanism models. (a) 3D-UNet + SE model prediction results. (b) 3D-UNet + CBAM model prediction results. (c) 3D-UNet model prediction results. (d) 3D-UNet + ECA model prediction results.

Moreover, this paper compares the prediction results among the proposed model and the other models in Xline, Inline, and Time slices, as shown in Figure 9.  Figure 9(a) shows the results of the proposed model; Figure 9(b) shows the results after adding the CBAM module to the 3D-UNet; Figure 9(c) shows the results only with the 3D-UNet; Figure 9(d) shows the results after adding the ECA module to the 3D-UNet. The 3D-UNet network only can identify faults but contain some fuzzy details, indicating a low recognition accuracy. After adding the SE module, the recognition results are better than adding other attention mechanism modules, and the results closely fit the labeled data.

**Figure 10:**
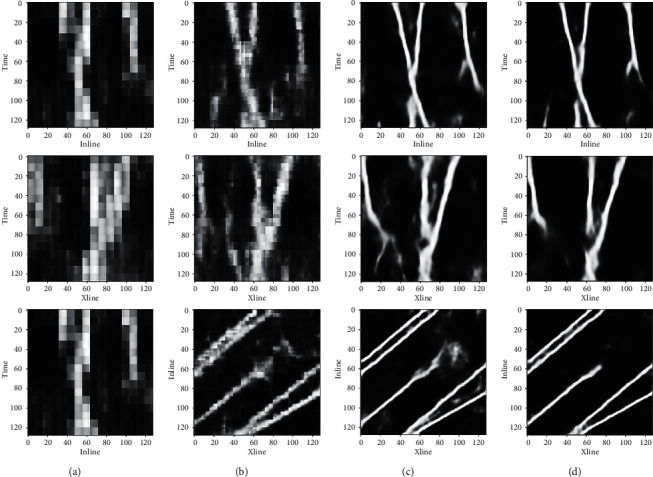
The recognition effects of different semantic segmentation models. (a) FCN8 model prediction results, (b) FCN4 model prediction results, (c) AttUNet model prediction results, and (d) UNet + SE model prediction results.

This paper also compares the different semantic segmentation models in Xline, Inline, and Time slices, as shown in Figure 10. Among the models, the FCN model has the lowest continuity and accuracy than the other models in the recognition results, as the FCN model upsamples directly with 8 and 4 strides, resulting in a checkerboard effect on the images. AttUNet is better than the FCN model but worse than the UNet model in terms of continuity and accuracy of recognition.

## 6. Conclusions

This paper transforms the fault recognition problem into a binary classification problem and proposes a new method to recognize faults using 3D-UNet and the attention mechanism of the neural network. Several conclusions can be drawn as follows:

1. The UNet architecture can effectively characterize the fault features in seismic data through encoding, decoding, and end-to-end training. Through skip connection to fuse feature information of multiple layers, this paper improved the accuracy and reliability of fault recognition.
2. The network uses the SE attention mechanism to capture channel attention through feature channels, which further improves the accuracy of network training. After multiple experiments, the loss function converges to 0.01, and the model accuracy rate is 94%. Compared with other attention mechanisms and network models, the proposed method can more accurately identify the fault location, enhance the generalization ability of the network structure, and reduce the artificial uncertainty of the fault interpretation results.
3. Due to the large size of the 3D data, the existing network architecture will lead to intensive computation. A combination of 3D volume and 2D slice can be considered in the future to train a more lightweight network architecture and achieve higher accuracy while having less computation.

## Figures and Tables

**Table 1:** The number of parameters of the compared attention mechanism.

Model	Parameters
3D-UNet [27]	1459585
3D-UNet-SE [34]	1634865
3D-UNet-CBAM [36]	1465515
3D-UNet-ECA [35]	1557873

**Table 2:** The number of parameters and FLOPs of the compared segmentation model.

Model	Parameters	GFLOPs
3D-UNet-SE [34]	1634865	2.72*e* + 02 G
FCN4 [37]	1503337	21.7 G
FCN8 [37]	1615689	21.6 G
AttUNet [38]	1756008	3.76*e* + 02 G

**Table 3:** Attention mechanism performance evaluation.

Indicator	Precision	Recall	F-1 score	AUC
SE [34]	**0.556**	0.942	**0.699**	**0.983**
CBAM [36]	0.440	**0.962**	0.603	0.979
3D-UNet	0.495	0.949	0.650	0.980
ECA [35]	0.548	0.936	0.691	0.982

**Table 4:** Compared models performance evaluation.

Indicator	Precision	Recall	F-1 score	AUC
FCN4	0.315	0.929	0.470	0.890
FCN8	0.236	0.879	0.371	0.940
AttUNet	0.504	**0.954**	0.660	0.982
UNet-SE [34]	**0.556**	0.951	**0.699**	**0.983**

## Data Availability

The network code is available from the corresponding author upon request. The data are available from https://github.com/xinwucwp.
